# RadioLab project: knowledge of radon gas in Italy

**DOI:** 10.1038/s41598-023-45809-6

**Published:** 2024-01-12

**Authors:** F. Ambrosino, G. La Verde, M. Colucci, V. Fanti, D. Barrale, A. Caciolli, S. Hemmer, M. L. De Giorgi, A. Ventura, J. Immè, A. Pagano, M. Budinich, M. Vascotto, V. Montalbano, M. Capua, R. Tucci, M. Chiosso, L. Visca, F. Groppi, M. Pugliese

**Affiliations:** 1 INFN-National Institute of Nuclear Physics, Napoli Section, Naples, Italy; 2 Department of Physics “Ettore Pancini”, University of Napoli Federico II, Naples, Italy; 3 INFN-National Institute of Nuclear Physics, Milano Section, Milan, Italy; 4 Department of Physics Aldo Pontremoli, University of Milano, Milan, Italy; 5 INFN-National Institute of Nuclear Physics, Cagliari Section, Cagliari, Italy; 6 Department of Physics, University of Cagliari, Cagliari, Italy; 7 INFN-National Institute of Nuclear Physics, Padova Section, Padua, Italy; 8 Department of Physics and Astronomy, University of Padova, Padua, Italy; 9 INFN-National Institute of Nuclear Physics, Lecce Section, Lecce, Italy; 10 INFN-National Institute of Nuclear Physics, Catania Section, Catania, Italy; 11 INFN-National Institute of Nuclear Physics, Trieste Section, Trieste, Italy; 12 INFN-National Institute of Nuclear Physics, Pisa Section, Pisa, Italy; 13 Department of Physical Sciences, Earth and Environment, University of Siena, Siena, Italy; 14 INFN-National Institute of Nuclear Physics, Cosenza Section, Cosenza, Italy; 15 Department of Physics, University of Calabria, Calabria, Italy; 16 INFN-National Institute of Nuclear Physics, Torino Section, Turin, Italy; 17 Department of Physics, University of Torino, Turin, Italy

**Keywords:** Applied physics, Environmental sciences, Environmental social sciences

## Abstract

RadioLab is an Italian project, addressed to school-age people, and designed for the dissemination of scientific culture on the theme of environmental radioactivity, with particular regards to the importance of knowledge of radon gas exposure. The project is a nationwide initiative promoted by the National Institute of Nuclear Physics- INFN. First tool used by the project, and of immediate impact to assess the public awareness on radon, is the administration of the survey “do you know the radon gas?”. In the survey, together with the knowledge of radon and of its sources, information on personal, cultural and territorial details regarding the interviewees are also taken. Reasonably, the survey invests not only young people, but also their relatives, school workers and, gradually, the public. The survey is administrated during exhibitions or outreach events devoted to schools, but also open to the public. The survey is in dual form: printed and online. The online mode clearly leads RadioLab project even outside the school environment. Based on the results of the survey, several statistical analyses have been performed and many conclusions are drawn about the knowledge of the population on the radon risk. The RadioLab benefit and the requirement to carry on the project goals, spreading awareness of environmental radioactivity from radon, emerge. The dataset involves all twenty Italian regions and consists of 28,612 entries covering the 5-year period 2018–2022.

## Introduction

Radioactivity Laboratory (RadioLab) is an Italian outreach project, carried on by the National Institute of Nuclear Physics (INFN) and designed to provide a tool that can be used at school level for scientific dissemination about environmental radioactivity^1, 2^. The project was born, more than a decade ago, from the consideration and observation that the human subjective perception of risk almost always does not coincide with the real risk, since everything that is not well known generates stress and anxiety, and, hence, perception of risk and danger. This aspect is particularly evident with the radioactivity issue. Strengthened by these assumptions, the basic idea of RadioLab is to bring the new generations (young people attending school), and indirectly the rest of the population, closer to the theme of ionizing radiation and the effects on human health^3^. This goal is achieved by several progressive activities, which include administration of surveys to assess the background knowledge, preparatory lessons on environmental radioactivity and its monitoring, laboratory sessions to carry out measurements, study sessions to handle the data with analysis and presentation of the results^4^. By means of these activities, school students are offered the opportunity to approach the world of scientific research, and become familiar with the use of detectors for ionizing radiation. Scientific communication, teaching and scientific research are integrated through the implementation of orientation training actions in a process that follows the phases of a scientific research work^5^. The activities carried out by the students within the project also promote the acquisition of transversal skills by developing their analytical skills, their ability to analyze and outline real situations, to manage information and to disseminate them^6^. The INFN sections directly involved in the RadioLab project are: Napoli, Milano, Cagliari, Cosenza, Lecce, Padova, Pisa, Torino, Trieste, Catania (until 2019). These sections collaborate with the regional schools, performing the goal activities, and also involving the population. Furthermore, RadioLab aims to spread in the regions close to the ones where the involved INFN sections are present. RadioLab pursues the common objective in all INFN sections that, however, have their own autonomy in declining the various activities of the project, according to the instrumentation at disposal and/or the particular local skills.

In the context of the RadioLab project, the issue of radioactive gas radon and its measurement is explored. Nowadays radon is highly topical, especially after the promulgation of the new Italian Legislative Decree 101/2020^7^ in the field of radioprotection, which is the transposition of the European Directive 2013/59/Euratom^8^. Radon (^222^Rn) is a radioactive natural gas, arising from the decay chain of ^238^U, which is present throughout the Earth’s crust. This gas is by far the most important source of ionizing radiation among the sources of natural origin, and radon is well-known to be the main cause of lung cancer just after cigarette smoking. Although RadioLab has been implemented for more than a decade, only recently (since 2018) a systematic monitoring activity about the knowledge of the problems related to radon exposure in closed environments has been introduced. The first activity used to assess the awareness on the radon issue is the administration of the survey “do you know the radon gas?”, in which questions about radon knowledge are proposed to the interviewees. The survey is intended not only for school-age people but also for their relatives, the school workers and the public; hence, an online version and a printed version exist, which are administered during exhibitions or outreach events. The results of the survey performed nationwide in Italy, together with a deep statistical analysis, are reported in this paper.

## Materials and methods

### Structure of the survey

Surveys are effective tools for gathering and synthesizing information in a compact way that can be easily managed for successive appropriate analysis^9^. On the other hand, surveys can be difficult to implement as a long time can be needed to get enough statistics. The survey “do you know the radon gas?” has been adopted to monitor the knowledge of radon gas in Italy, and its structure is reported in Table 1: the knowledge of radon and of its sources together with information on personal, cultural and territorial details of the interviewees are catched. In particular, the questions are: (1) ‘date of the survey’, used to obtain a trend on the radon knowledge over the years (from 2018 to 2022); (2) ‘scenario where the survey was done’, used to obtain information about the dissemination activity (event; online; school/university); (3) ‘home city of the interviewee’, used to map the data; (4) ‘gender of the of interviewee’, used to evaluate the distribution of the survey between males and females; (5) ‘age of the of interviewee’, used to evaluate the distribution of the survey among school population (< 19), university age range (19–30), adulthood range (30–50), adult and senior population (> 50); (6) ‘education level of the interviewee’, used to correlate the knowledge and background on the radon issue; (7) ‘do you know radon?’, i.e. the main question of the survey about the knowledge of radon gas; (8) ‘if you know radon, please add the knowledge source’, used to establish the way in which the knowledge of the radon issue is achieved and to evaluate the effectiveness of RadioLab; (9) ‘do you think a radon measurement in your city is urgent?’, used to evaluate the risk perception of radon. The survey is in dual form: online (web.infn.it/RadioLAB/) and printed. It is administered during exhibitions or outreach events, summer/spring workshops, dissemination events like the European Researchers' Night and the European Radon Day, and seminars in universities and schools^10, 11^. These modalities are mainly addressed to school-age population, but the implementation of such methodology also leads to the spread of the survey even outside the school environment. In fact, students continue to administer the survey interviewing other peers, family members, friends, and acquaintances. In this regards, the online version of the survey is of great help, allowing to carry it out comfortably from home, at work or anywhere. A QR-code has also been generated, allowing the easy compilation of the survey on the smartphones. The administration of the survey is approved by the INFN and the informed consent was obtained from all involved subjects.

**Table 1:** Schematic view of the survey “do you know the radon gas?” within the RadioLab project.

Questions of the survey “do you know the radon gas?”	Possible answers
Date of the survey	From 2018 to 2022
Scenario where the survey was done	Event; online; school/university
Home city of the interviewee	Italian locations
Gender of the of interviewee	Male; female
Age of the of interviewee	< 19; 19–30; 30–50; > 50
Education level of the interviewee	Primary/middle school diploma; high school diploma; university degree
Do you know radon?	Yes; no
If you know radon, please add the knowledge source	Project; newspaper/tv/web; event; other
Do you think a radon measurement in your city is urgent?	Yes; no; I do not know

All questions and the corresponding possible answers are reported. The questions are the same for the online and the printed versions of the survey.

### Data management and analysis

The total number of collected surveys are 29,106. About the 1.7% of the data (494) had anomalies linked to the incorrect filling of the survey by interviewees, for instance interviewees with age < 19 stating to have university degree, or interviewees saying they do not know what radon is but add newspaper/tv/web as source of knowledge. These inconsistent 494 surveys have been excluded. The final analysed dataset consists of 28,612 entries, covering the five-year period 2018–2022 and involving all twenty Italian regions. On these data, both qualitative and quantitative statistical analyses have been performed. The qualitative description of the dataset reports: (i) the number of surveys per region; (ii) the amount of male and female involved, grouped by age and education level; (iii) the number of survey sorted by ‘scenario where the survey was done’; (iv) the number of surveys per year. The quantitative analysis of the dataset reports several bar graphs, combining together two and/or three questions of the survey. The first set shows bar graphs of the answers from the main question ‘do you know radon?’ matched with: (i) the gender and age of the interviewees; (ii) the education level of the interviewees; (iii) the age of the interviewees and the source of their knowledge if they know what radon gas is. The second set shows bar graphs of the answers from the question ‘do you think a radon measurement in your city is urgent?’ matched with: (i) the age of the interviewees; (ii) the education level of the interviewees. The quantitative statistical analysis is concluded by: (i) the bar graph of the answers from the main question ‘do you know radon?’ matched with the answers from the question ‘do you think a radon measurement in your city is urgent?’; (ii) the trend per age of the interviewees who know radon during the years of administration of the survey (2018–2022).

### Human ethics

All experiments were performed in accordance with the current and relevant guidelines and regulations in in force; the administration of the survey was approved by the INFN; the informed consent was obtained from all involved subjects.

## Results and discussion

### Qualitative analysis of the data

**Figure 1:**
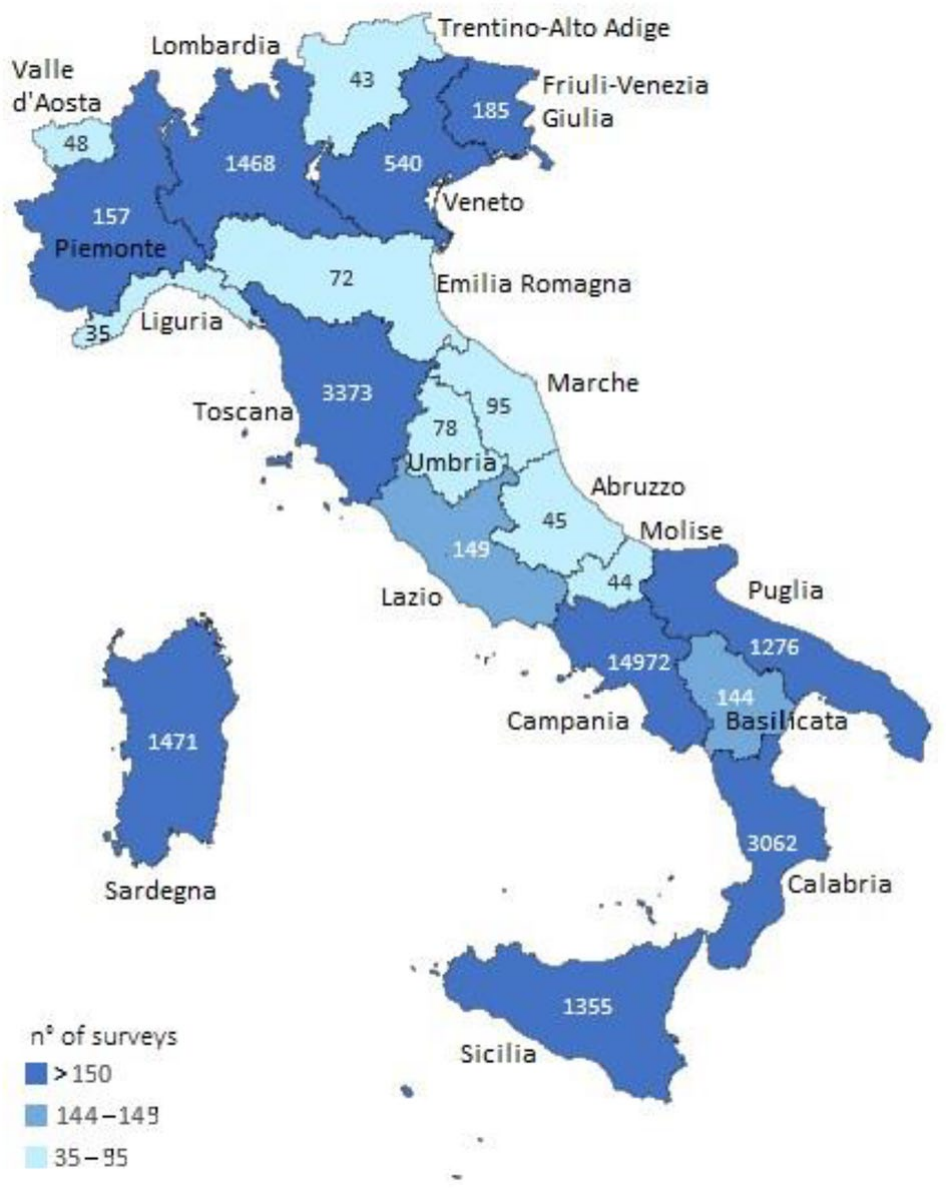
Number of surveys “do you know the radon gas?” carried out by region. The map was created by Microsoft Excel 2016 version number 16.78 (https://office.microsoft.com/excel).

The ten Italian regions in dark blue in Fig. 1 (Campania, Lombardia, Sardegna, Calabria, Puglia, Veneto, Toscana, Piemonte, Friuli-Venezia Giulia, Sicilia), i.e. those with more than 150 surveys administered, correspond to the ten regions in which the INFN sections directly involved in the RadioLab project (Napoli, Milano, Cagliari, Cosenza, Lecce, Padova, Pisa, Torino, Trieste, Catania) are located. The regions in light blue in Fig. 1 (Valle d’Aosta, Trentino-Alto Adige, Liguria, Marche, Emilia-Romagna, Umbria, Abruzzo, Molise), in which no INFN sections are directly involved, carried out the administration of less than 100 surveys. This aspect demonstrates the spread of RadioLab in dissemination of scientific culture on the theme of environmental radioactivity, in particular related to the knowledge of radon gas. It is interesting to note the case of the two regions in sky-blue in Fig. 1, which, with 144 (Basilicata) and 149 (Lazio) surveys administered, are affected by a greater influence due to the neighboring regions in which INFN sections are present. Campania region administered the highest number of surveys (14,972), followed at a considerable distance by Toscana (3373) and Calabria (3062).

**Figure 2:**
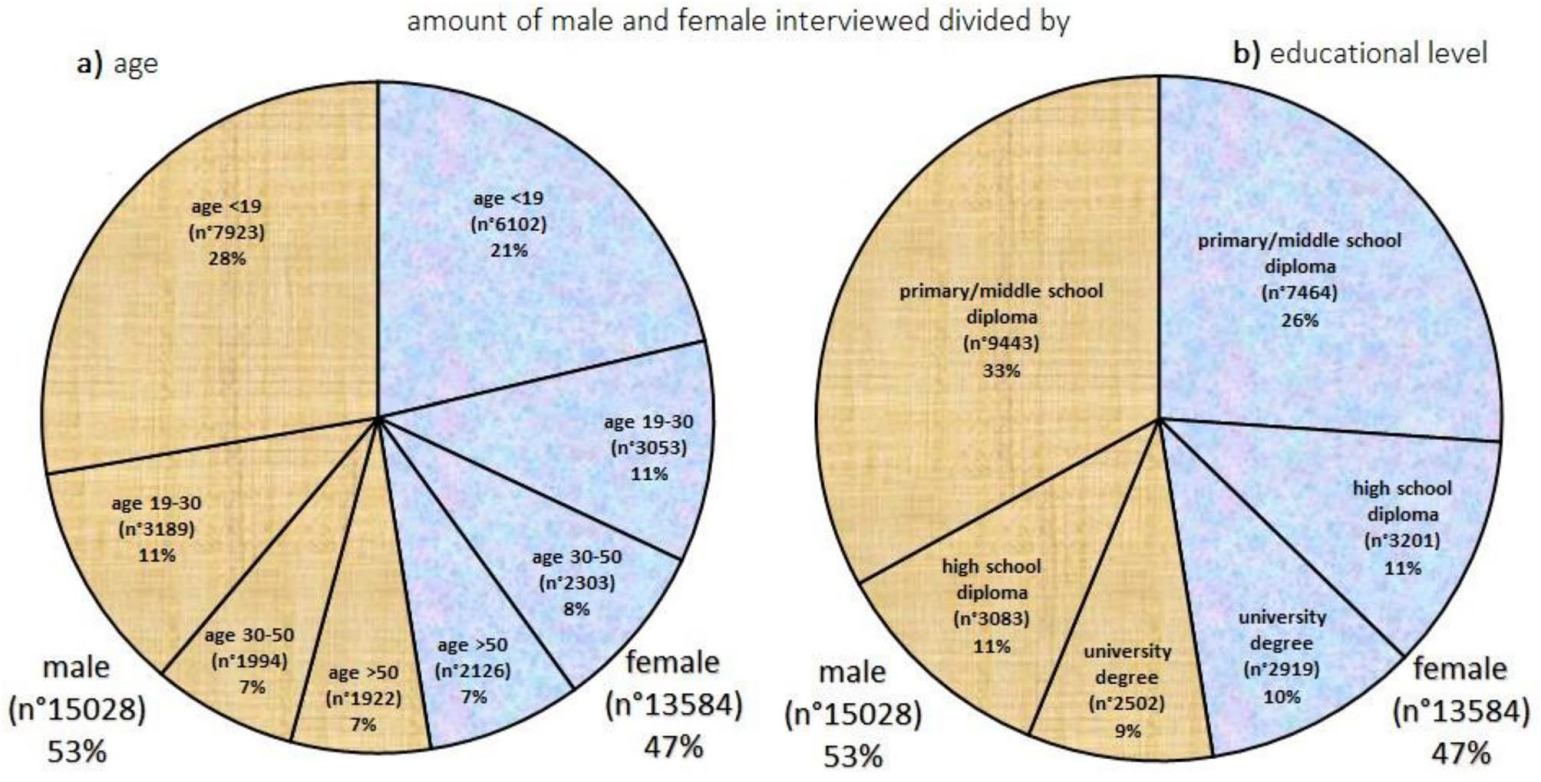
Number and percentage of male (wood color) and female (jeans color) to which survey “do you know the radon gas?” was administered, assorted by: (**a**) age of the interviewees (< 19; 19–30; 30–50; > 50); (**b**) education level of the interviewees (primary/middle school diploma; high school diploma; university degree).

From Fig. 2 is evident that an almost perfect gender balance is present, both among the various age groups and among the education level groups. The highest percentage of interviewees (49%) regards young people (< 19), and it decreases as the age increases (Fig. 2a). This aspect is due to the fact that RadioLab is mainly designed for school-age interviewees, and it is confirmed, as can be seen in Fig. 2b, by the fact that primary/middle school diploma is the most frequent education level (59%). The amount of the interviewees decreases with the increase in the education level (Fig. 2b).

**Figure 3:**
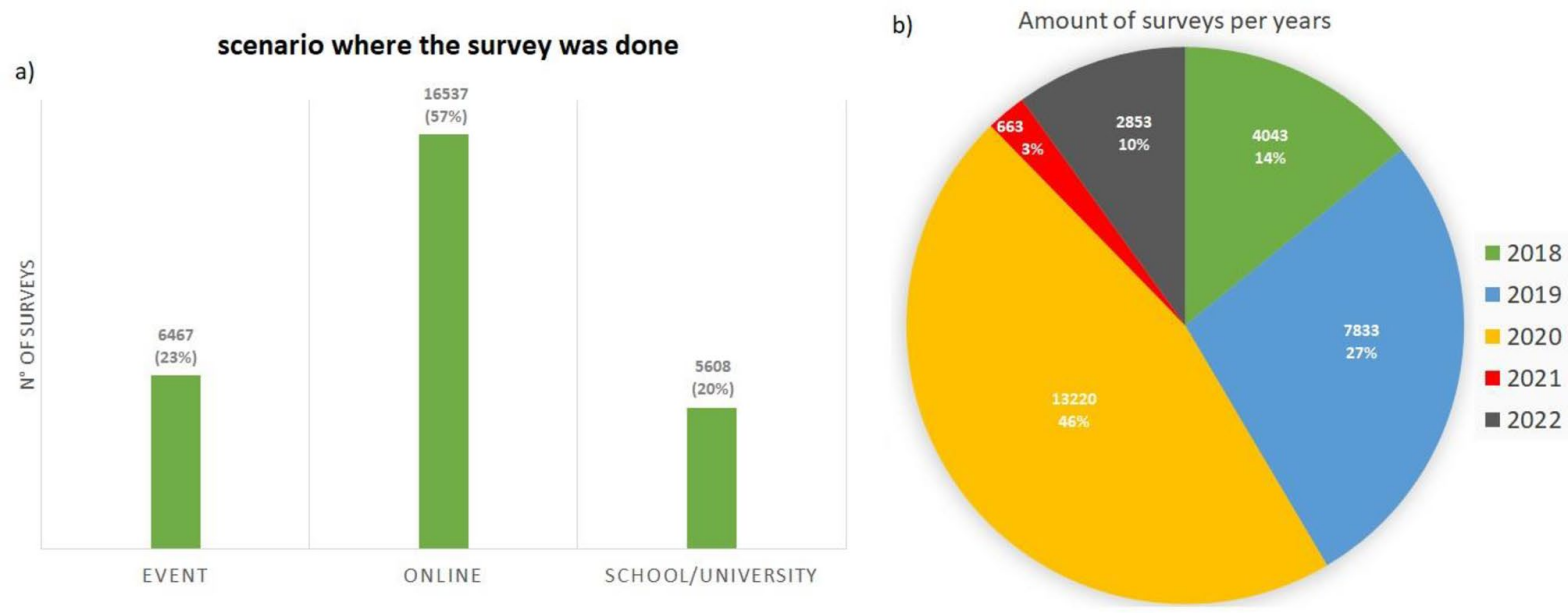
Number and percentage of survey “do you know the radon gas?” slitted by: (**a**) scenario where the survey was done (event; online; school/university), (**b**) years (2018–2022).

The survey “do you know the radon gas?”, being the easiest way, has been carried out mostly in online mode (57%), then at scientific events (23%) and at schools/universities (20%) during seminars (Fig. 3a). In the time frame of the administration of the survey “do you know the radon gas?”, most of the entries (46%) have been gathered in the year 2020. Such high value is actually explicable by the promulgation in 2020 of the D.lgs 101^7^ implementation of the 2013/59/Euratom^8^, which establishes the basic safety standards relating to protection against the risk arising from exposure to ionizing radiation. In particular, the radon exposure topic is addressed by proposing a national radon action plan and fixing a reference level of 300 Bq/m^3^. With the D.lgs 101/2020, a great attention of public opinion has been given to radon issue, and, thus, this fact explains the increase in filling up the survey “do you know the radon gas?” in 2020^12^. The early years 2018 (14%) and 2019 (27%) were also productive, testifying the growing interest in radon issue (Fig. 3b). The years 2021 (3%) and 2022 (10%) show a notable decrease in performing the survey, mainly due to the peak period of the COVID-19 pandemic^13^ (Fig. 3b).

**Figure 4:**
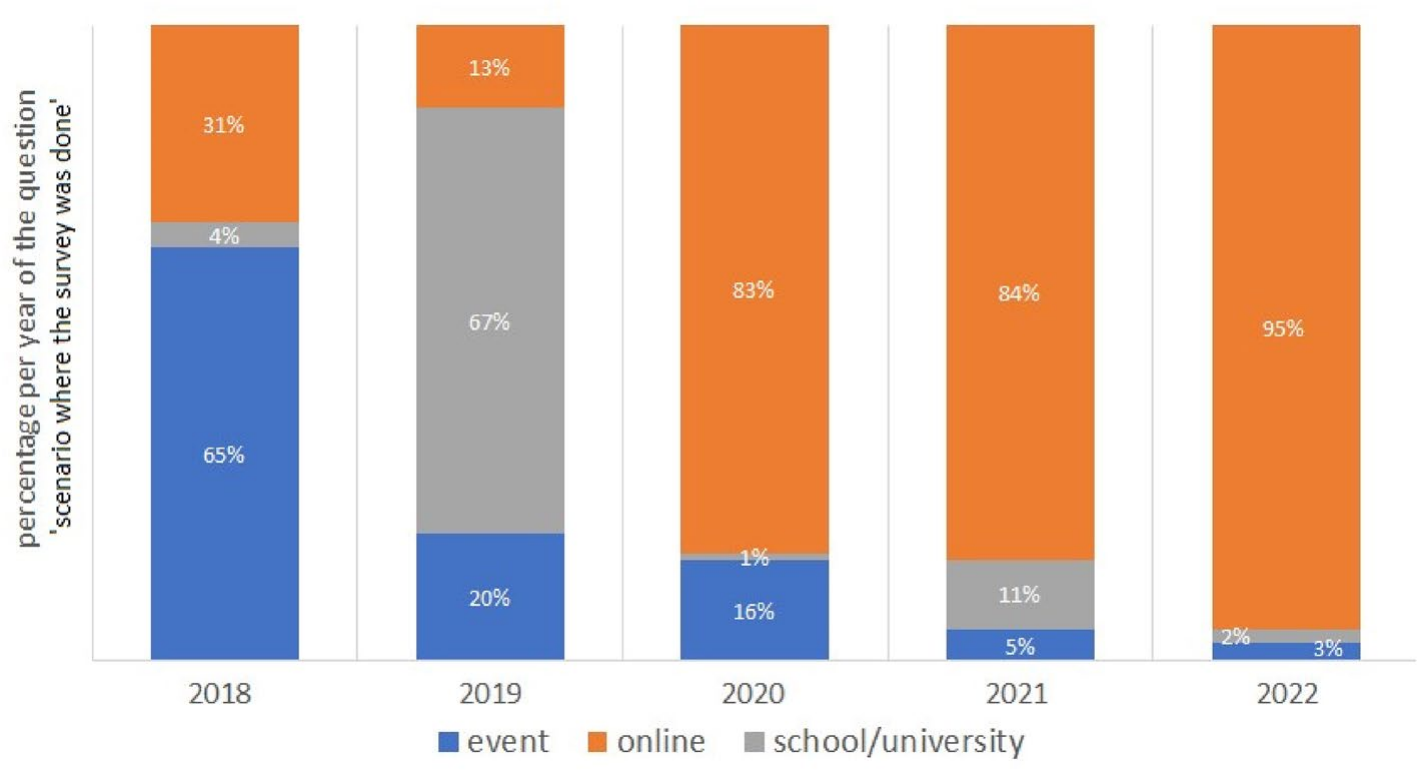
Percentage per year (2018–2022) of the question ‘scenario where the survey was done’ (event in blue; online in orange; school/university in gray), concerning the survey “do you know the radon gas?”.

A more detailed analysis has been done in Fig. 4 concerning the percentage per year of question ‘scenario where the survey was done’. Firstly, Fig. 4 highlights the in-presence modalities (events and at schools/universities) for carrying out the survey as the main ways used in the initial years, before the COVID-19 disease: 2018 with 69%, increasing in the 2019 with 87%. From 2020 to 2022, due to COVID-19, the predominant mode for carrying out the survey is the online one, with about 83% in 2020 and 2021, up to 95% in 2022^13^.

Overall qualitative analysis denotes the statistically significance of the used sample.

### Quantitative analysis of the data

**Figure 5:**
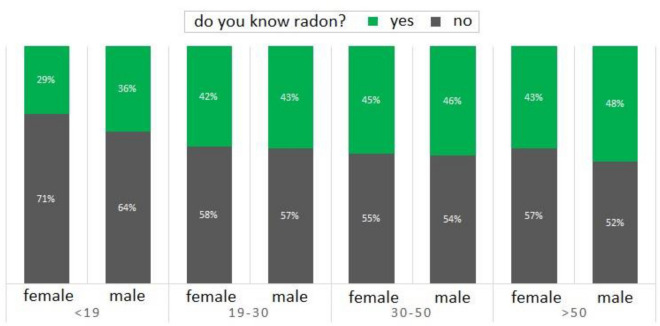
Bar graph of the percentage per gender about the knowledge of radon gas (‘yes’ in green, ‘no’ in black) combined with the age of the interviewees (< 19;19–30;30–50; > 50).

A deep quantitative statistical analysis on the 28,612 data resulting from the survey “do you know the radon gas?” is presented in the following. In total, 39% of interviewees declare to know radon gas. Figure 5 reports the percentage per gender of the interviewees who know or do not know the radon gas, combined with the age. This analysis underlines several aspects: (i) the male are more informed about radon (male 40% and 37% female, on average); (ii) the difference between male and female about the knowledge of radon is slight in the age groups of 19–30, 30–50, > 50 (~ 3%), while it is more marked in the age < 19 (7%); (iii) the knowledge of radon is nearly constant in the age groups of 19–30, 30–50, > 50 (~ 45%), with a clear decrease in younger age < 19 (~ 32%)^14^.

**Figure 6:**
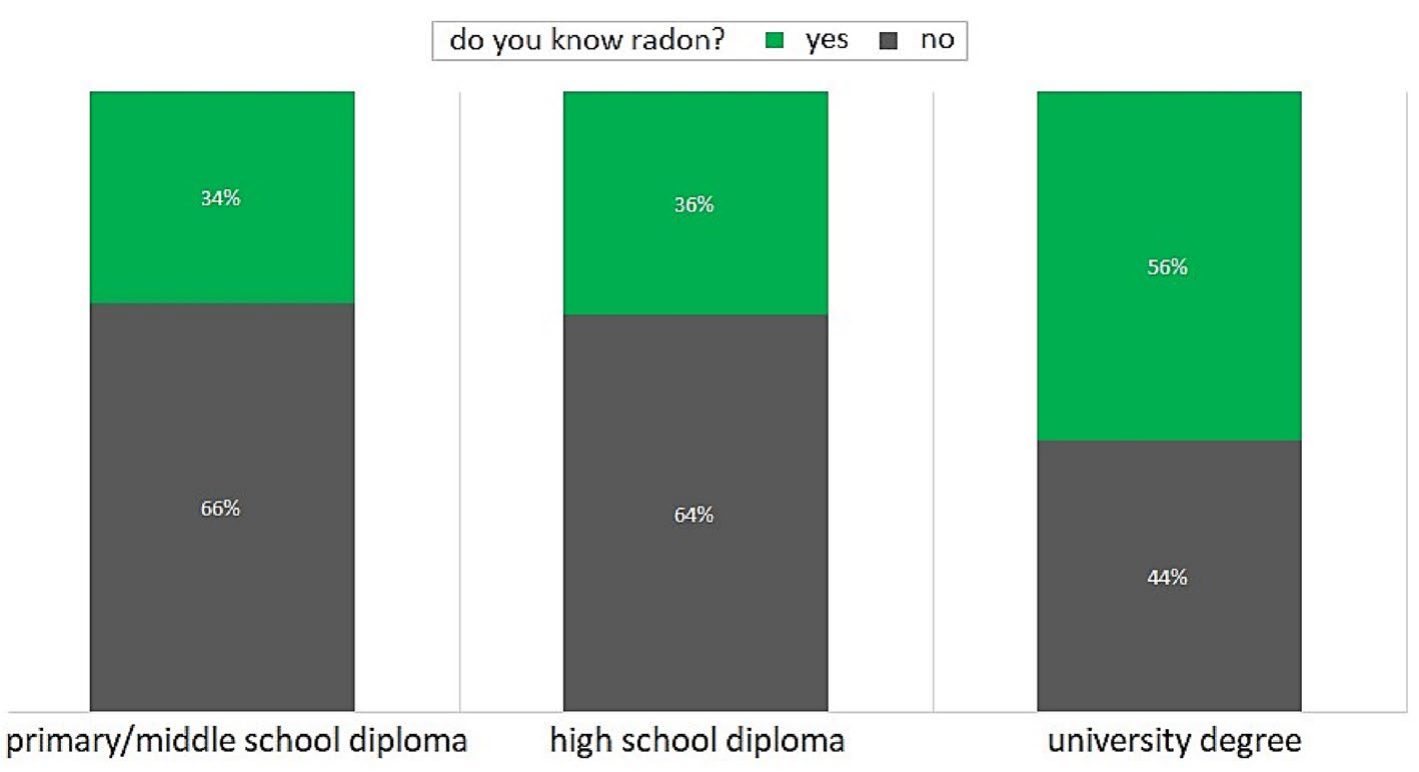
Bar graph of the percentage per education level (primary/middle school diploma; high school diploma; university degree) on the knowledge of radon (‘yes’ in green, ‘no’ in black).

Figure 6 reports the percentage per education level of the interviewees who know or do not know the radon gas. This analysis highlights the evident influence of the education level in the knowledge of radon issue: the interviewees who know radon grow with increasing education level^15^. In particular: (i) the number of interviewees who know radon having high school diploma is slightly higher than the one of those having primary/middle school diploma (difference of 2%); (ii) most of interviewees having the highest level of education, i.e. university degree, know radon (56%), exceeding the two lower levels of education by more than 20%.

**Figure 7:**
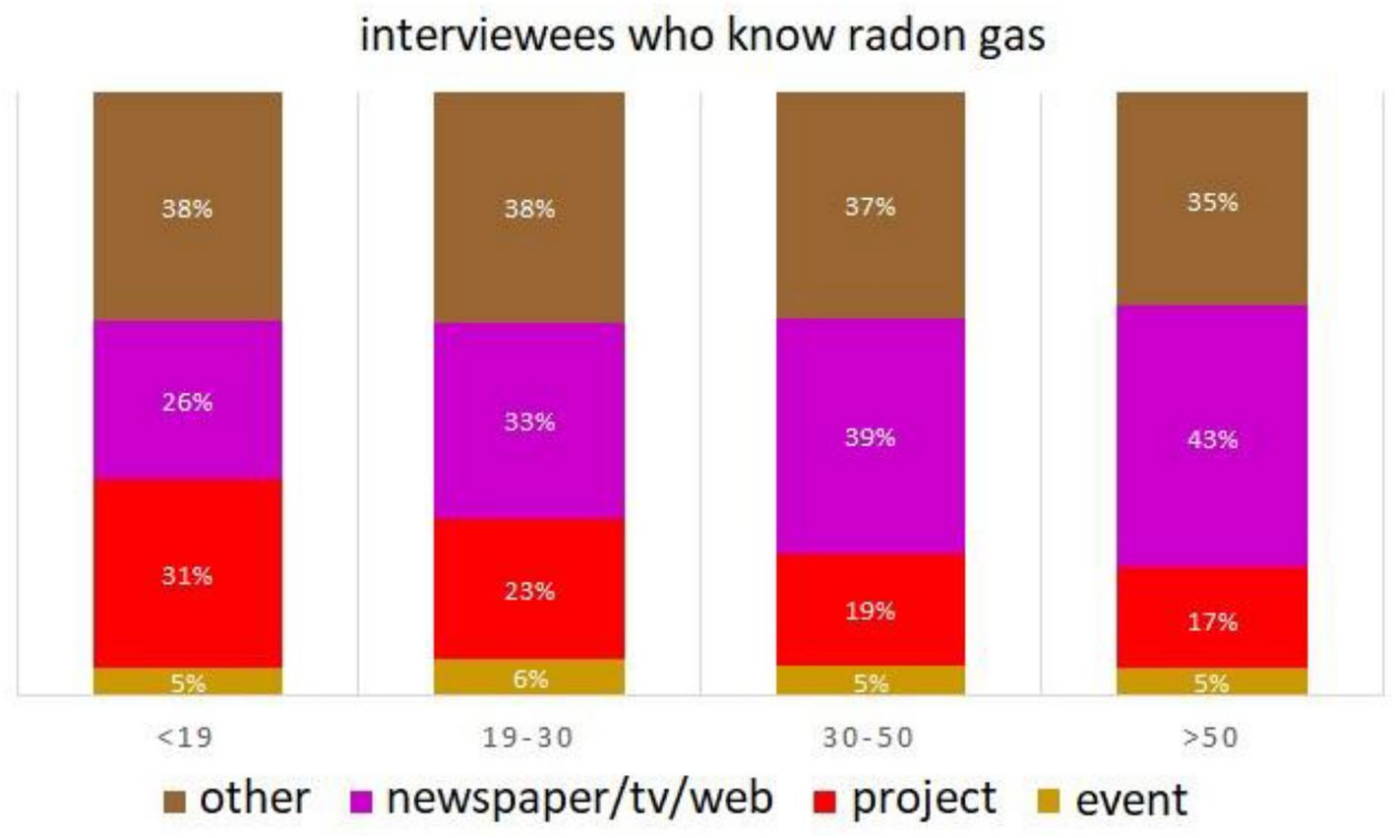
Bar graph of the percentage per source of radon knowledge (project in red; newspaper/tv/web in violet; event in ocher; other in brown) for interviewees, combined with their age (< 19;19–30;30–50; > 50).

Figure 7 reports the percentage per source of radon knowledge for interviewees who declare to know the gas, combined with their age. This analysis reveals that interviewees who know radon gas: (i) from projects decrease with the age increase (from 31 to 17%); (ii) from newspapers/tv/web increase with the age increase (from 26 to 43%); (ii) from exhibitions or orientation events and other sources are costant (~ 5% event, ~ 37% other). Projects are mostly widespread in the school-age population (< 19), indeed RadioLab is mainly designed for students and raised awareness in the theme of radon gas. Older people mention newspapers/tv/web as the most popular media source of knowledge of radon gas.

**Figure 8:**
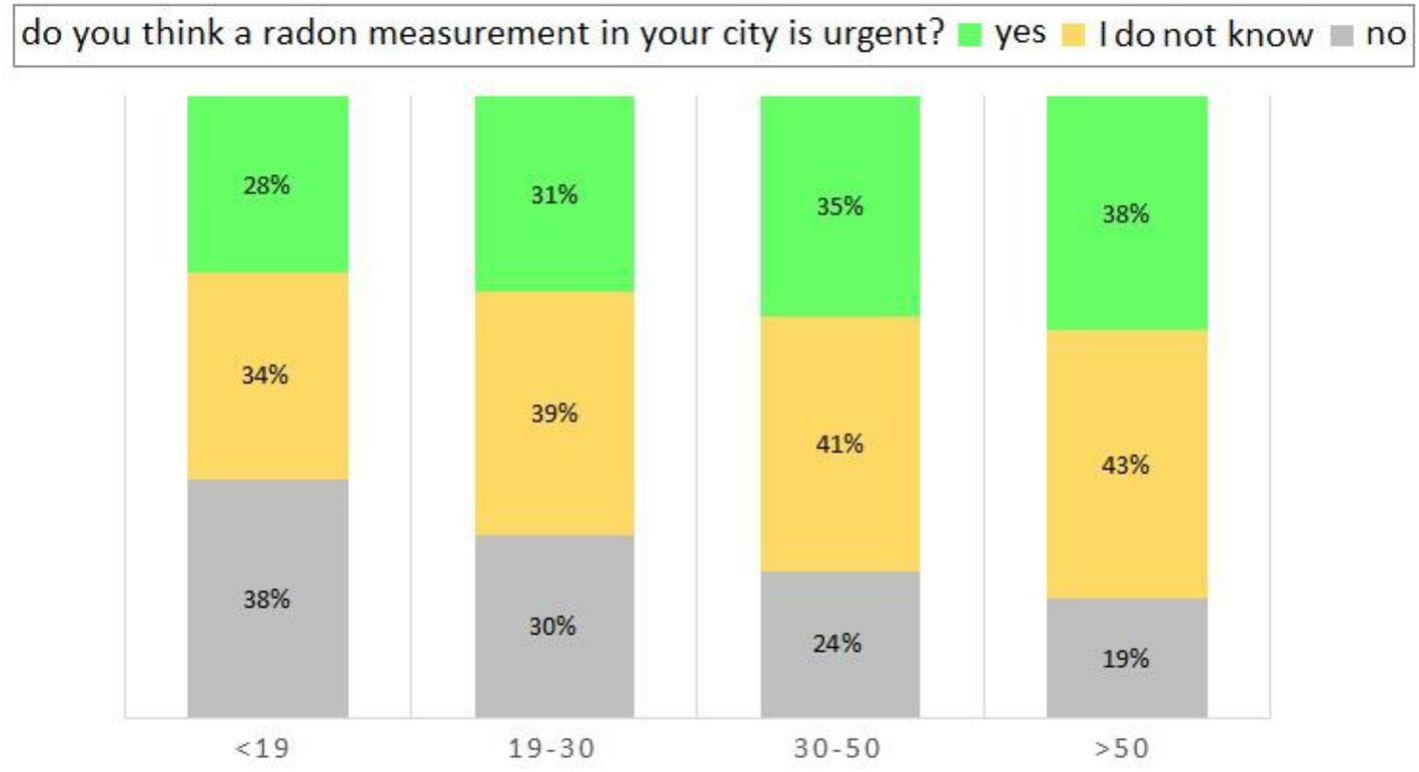
Bar graph of the percentage per age of the interviewees (< 19;19–30;30–50; > 50) answering the question ‘do you think a radon measurement in your city is urgent?’ (‘yes’ in lime; ‘I do not know’ in gold; ‘no’ in silver).

Figure 8 reports the percentage per age of the interviewees who consider the three options for a radon measurement in their city: urgent, not urgent, do not know. This analysis shows: (i) deeming it urgent, or not knowing whether it is urgent increases as respondents age; (ii) deeming a radon measurement in their city not urgent decreases as the age of interviewees increases. Awareness of radon issue increases in adulthood, being aware about the potential risk of radon^14^.

**Figure 9:**
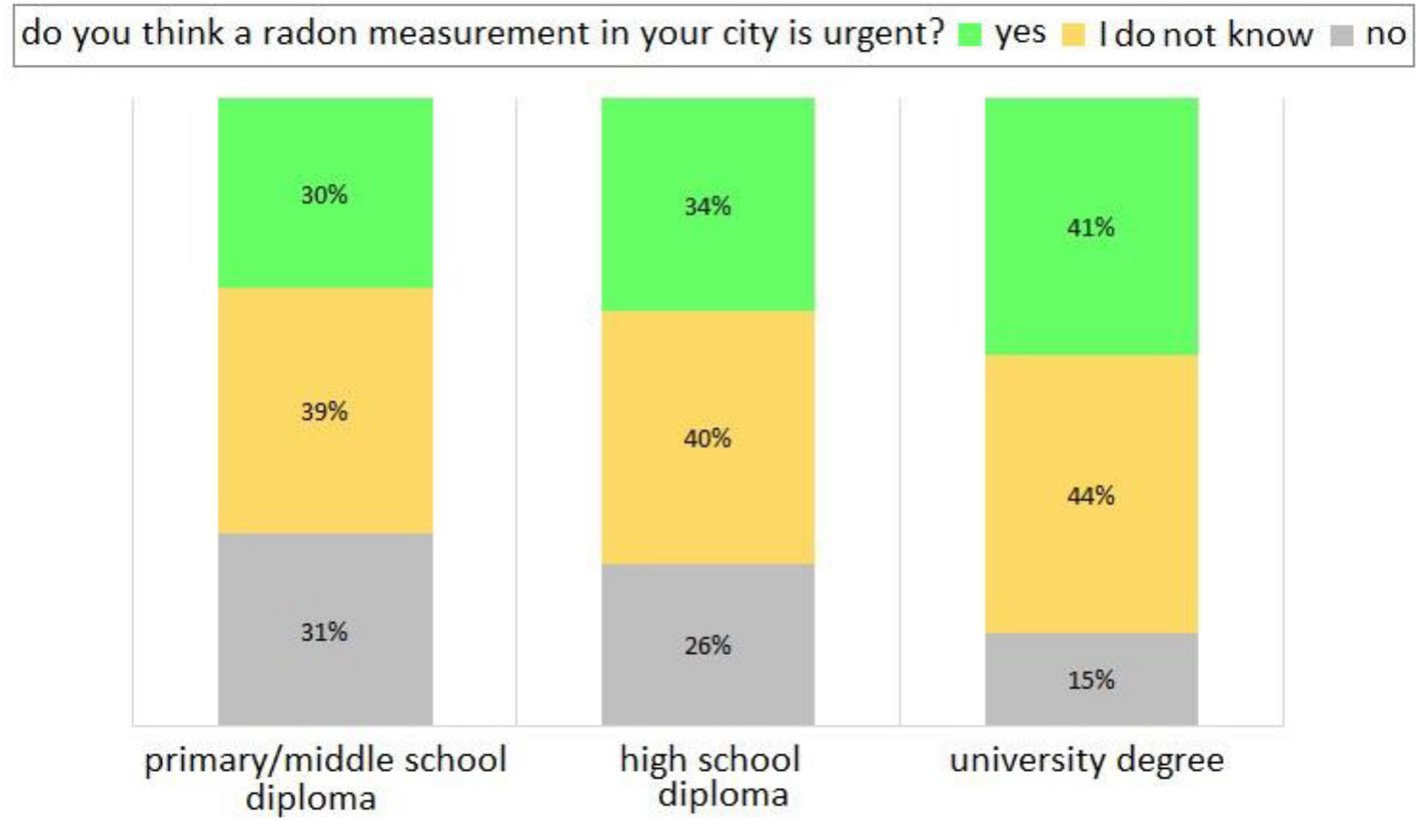
Bar graph of the percentage per education level of the interviewees (primary/middle school diploma; high school diploma; university degree) answering the question ‘do you think a radon measurement in your city is urgent?’ (‘yes’ in lime; ‘I do not know’ in gold; ‘no’ in silver).

Figure 9 reports the percentage per education level of the interviewees who consider the three options for a radon measurement in their city: urgent, not urgent, do not know. This analysis highlights: (i) deeming it urgent, or not knowing whether it is urgent increases as the education level of interviewees; (ii) deeming a radon measurement in their area not urgent decreases as the education level of interviewees increases. Similar considerations drawn from Fig. 8 can be transposed to Fig. 9, showing that the awareness of radon issue increases with the education level. Joining together the results from Figs. 8 and 9, it emerges that the knowledge of the problems related to radon exposure in environments is strongly linked to the education level of the people, and hence, to their age^14, 15^. However, it is interesting to note that the percentage of people who do not consider urgent a radon measurement in their city is always less than a half of each group of interviewees by age and by education level.

**Figure 10:**
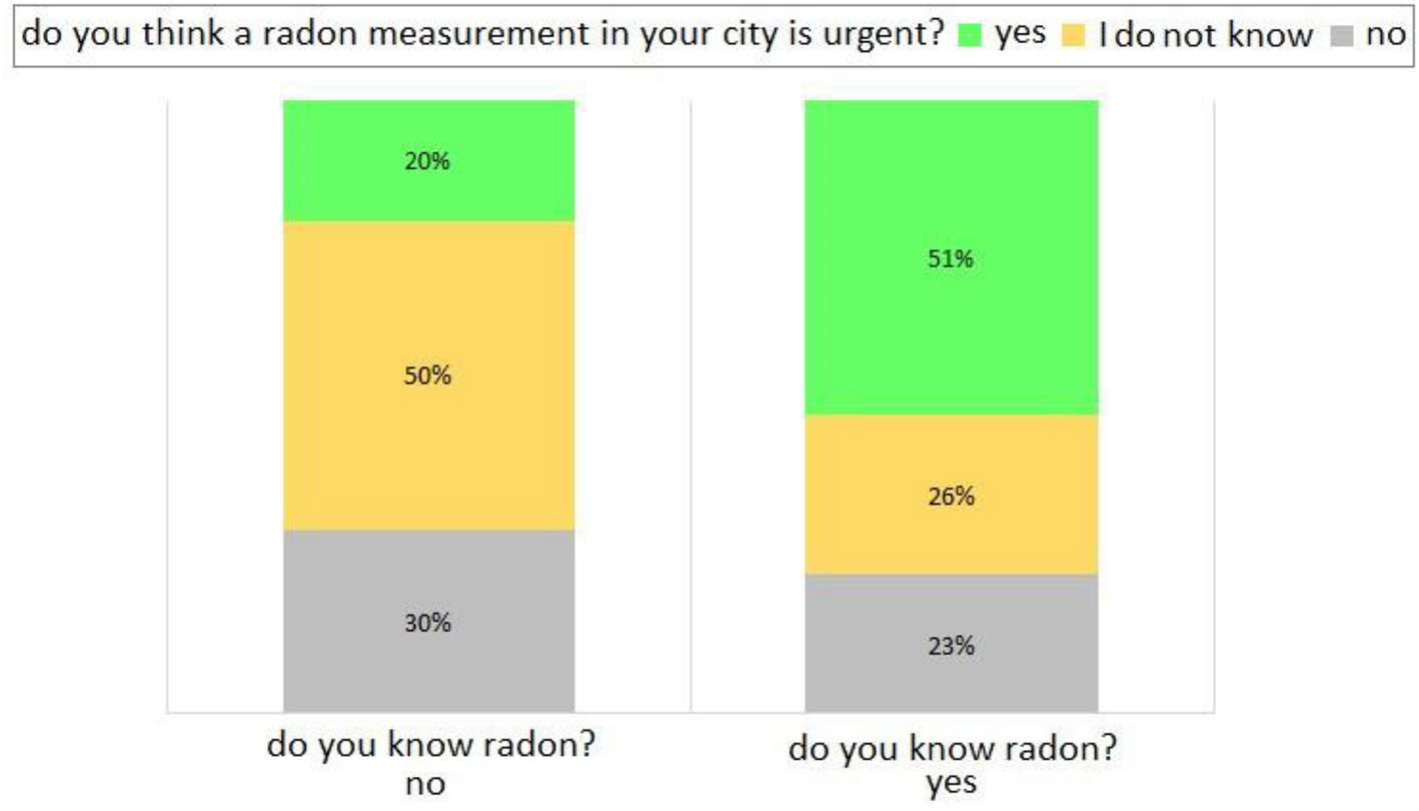
Bar graph of the percentage per interviewees who know or do not know about the radon gas, answering the question ‘do you think a radon measurement in your city is urgent?’ (‘yes’ in lime; ‘I do not know’ in gold; ‘no’ in silver).

**Figure 11:**
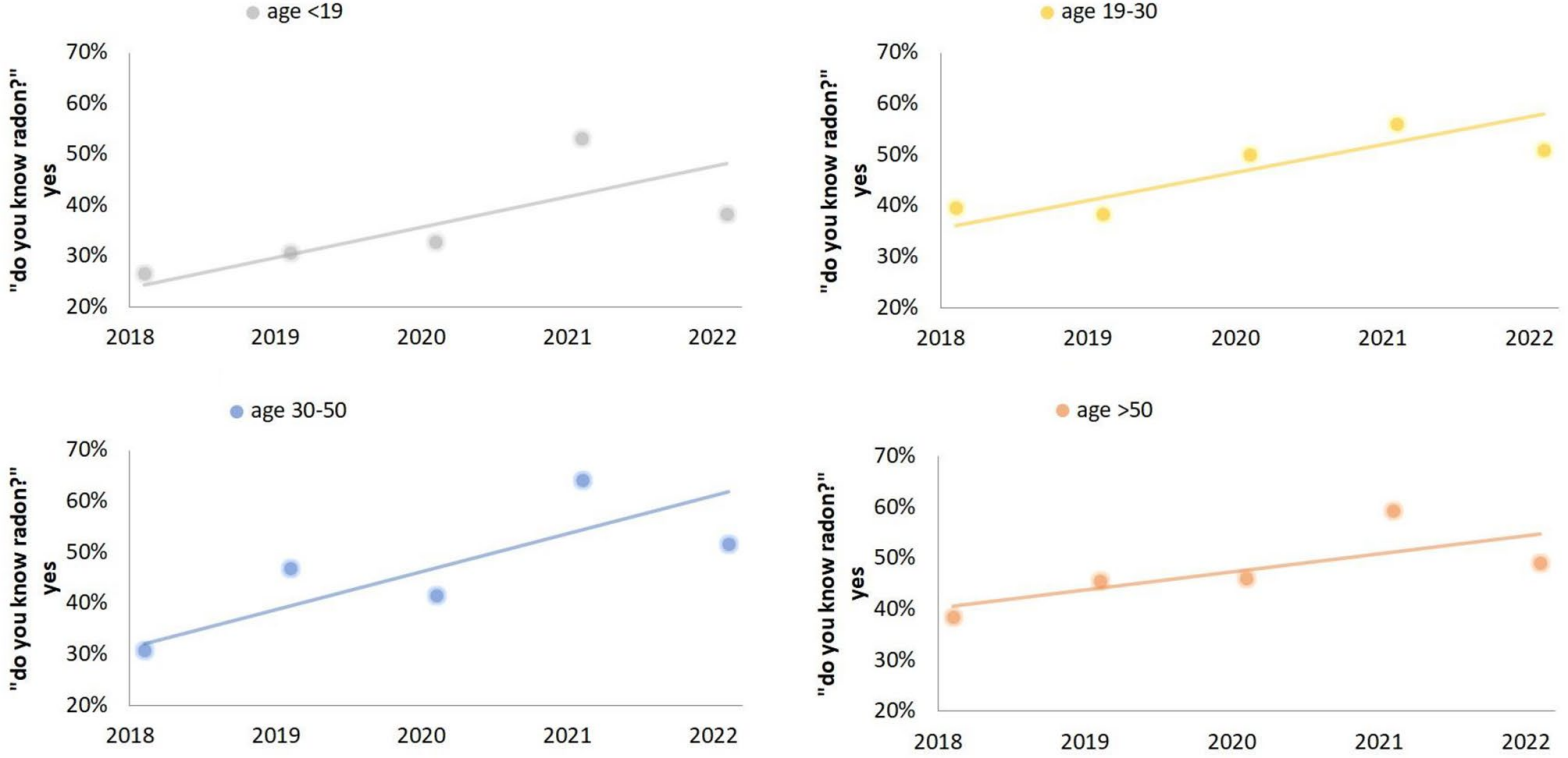
Trend per age (< 19 with gray line; 19–30 with yellow line; 30–50 with blue line; > 50 with orange line) of the interviewees who answered ‘yes’ to the question ‘do you know radon?’, during 2018–2022.

The last part of the quantitative statistical analysis is reported in the last two figures, i.e. Figures 10 and 11. Figure 10 represents the bar graph of the answers to the main question ‘do you know radon?’, matched with the answers from the question ‘do you think a radon measurement in your city is urgent?’. Most of the interviewees familiar with the topic of radon reply that gas measurement is urgently needed (51%), while the minority of the interviewees is divided almost equally between those who doubt and those who are not concerned about the radon measurement. The situation is completely different when the interviewees are those who are not familiar with the radon issue: (i) half of them is unsure whether or not a measure of radon in their city is urgent (50%); (ii) the other half is divided into 30% of people who do not want to measure radon in their city, and 20% who want to measure it. This is significant of the fact that the knowledge of a topic, whatever it may be (in this case radon), increases awareness of it and everything related to it (in the case of radon: risk perception), while ignorance on a topic tipically generates insecurity and uncertainty^16^.

Figure 11 represents the trend by age of the interviewees about the knowledge of radon during the years of administration of the survey “do you know the radon gas?”, from 2018 to 2022. The knowledge of radon increases over the years, mainly in interviewees in school-age with a percentage gain of 30% in 2021. This figure demonstrates the real effectiveness of the scientific dissemination on the radon theme promoted by RadioLab, mainly for the students for which the project was designed. In the age group 19–30, and especially in adults 30–50 and > 50, the increase in knowledge of radon can also be associated with the promulgation of D.lgs 101/2020^7^, which establishes the basic safety standards related to protection against the dangers arising from exposure to ionizing radiation, and in particular to the radon exposure (“Qualitative analysis of the data” and Fig. 4).

## Conclusions

As one of the most important tool for collecting and synthesizing information in a compact and easy way for data analysis, the survey is used in this paper to assess the awareness on radon gas. The survey “do you know the radon gas?” was administered to school students mainly, but also to the public, as part of the national RadioLab project (promoted by INFN), aimed at disseminating scientific culture with particular regard to the theme of radioactivity. This survey provides a state of the art on the radon gas knowledge in Italy since 2018, and how this knowledge has been changing over the years until 2022. The survey includes some questions about personal, cultural and territorial information of the interviewees, and the main questions about radon knowledge and the source of this knowledge, and, finally, a question about the need for radon measurements in the city of the interviewees. The survey has been administered both online and in printed version during scientific or orientation events, workshops and seminars held in universities and schools. The main results on a statistically significant sample of 28,612 interviewees, during the 2018–2022 period, highlight: (i) the knowledge of radon increases with the age and the education level of the interviewees, as does the awareness of considering the radon measurement in their city urgent; (ii) knowing radon also means knowing the risk it entails and, therefore, the need to monitor the gas; (iii) the real effectiveness of the RadioLab project in raising the knowledge of radon in the years investigated, especially among school students. These results are independent of gender since no particular differences are found between male and female answers. RadioLab is still ongoing, aiming to increase the number of surveys to be administered in the Italian regions with fewer numbers, in order to make familiar and raise awareness of more and more students, teachers, relatives and general public about the presence of environmental radioactivity of natural origin, in particular radon gas and its risks.

## Data Availability

The datasets used and/or analysed during the current study available from the corresponding author on reasonable request.
